# A complete, homozygous *CRX* deletion causing nullizygosity is a new genetic mechanism for Leber congenital amaurosis

**DOI:** 10.1038/s41598-018-22704-z

**Published:** 2018-03-22

**Authors:** M. T. Ibrahim, T. Alarcon-Martinez, I. Lopez, N. Fajardo, J. Chiang, R. K. Koenekoop

**Affiliations:** 1 Department of Paediatric Surgery, Human Genetics, Ophthalmology and McGill Ocular Genetics Laboratory, McGill University Health Center Research Institute, Montreal, Quebec, Canada; 2 Molecular Vision, Hillsboro, OR USA

## Abstract

*CRX* is a transcription factor required for activating the expression of many photoreceptor-neuron genes. *CRX* may be mutated in three forms of human blindness; Leber congenital amaurosis (LCA), cone-rod degeneration (CRD) and retinitis pigmentosa (RP). The pathogenic mechanism in most cases is likely dominant negative, with gain of function. We report a novel, complete homozygous *CRX* deletion in LCA. We identified a Lebanese family with 3 affected LCA cases. The proband was sequenced by NGS. Quantitative PCR, array comparative genomic hybridization, and long range PCR were performed. Full eye examinations, OCT and photography were performed. We identified a homozygous 56,000 bp deletion of *CRX*, which co-segregates and is heterozygous in four parents, who report normal vision. The blind children with LCA manifest severe retinal degeneration, a phenotype typical for *CRX* and LCA. We hypothesized that a single copy of *CRX* (haplo-insufficiency) in the causes mild abnormal foveal development, but not LCA. Two parents had significant inner and outer foveal and photoreceptor abnormalities. This is the first reported case of a homozygous, complete CRX deletion. Nullizygosity of *CRX* thus causes LCA while haplo-insufficiency of *CRX* causes abnormal foveal development, but not LCA. Our data suggest a new disease mechanism for *CRX*.

## Introduction

*CRX* encodes a 299 amino acid, homeodomain containing, transcription factor named **c**one-**r**od homeobo**x** gene (MIM #602225). In retinal progenitor cells (RPCs), CRX is inhibited by PAX6 and thereby prevents premature activation of photoreceptor differentiation. CRX is thus required for terminal differentiation and survival of photoreceptors and is one of the earliest selective markers of photoreceptor precursors.

The mature vertebrate retina is composed of six major neurons and one type of glial cell (Müller cell), which are organized into three cellular layers and two synaptic layers (plexiform layers): retinal ganglion cells are in the top layer (GCL); horizontal, amacrine, and bipolar interneurons, and Müller cells are in the middle layer, forming the inner nuclear layer (INL); and finally cone and rod photoreceptors are in the photoreceptor layer or bottom layer, the outer nuclear layer (ONL)^1,2^. The inner and outer plexiform layers (IPL and OPL) are the synaptic layers comprising the respective intermediate layers. During retinogenesis, these seven cell types arise from a common population of retinal progenitor cells (RPCs) in an evolutionarily conserved, precisely tuned, temporal birth order. In the center of the retina a special, unique circular region develops in some animals for high acuity vision, called the fovea.

Foveal development characterizes humans, apes and several other species, but is not universally present in the animal kingdom. Despite its importance, foveal development is still poorly understood. Hendrickson and her group have documented the retinal histological and *in vivo* microscopic changes in the human fovea from prematurity to adulthood^3^. They documented that foveal development commences *in-utero* with the formation of a pit at fetal week 11^3^, but continues long after birth^3^. The fovea is characterized by being cone rich, rod-free, and avascular. Notable changes are the centrifugal (outwards) migration of the top three inner retinal layers the GCL, INL and IPL, umbo (pit) formation, cone packing, and the formation of the foveal avascular zone (FAZ)^3^. The molecular determinants of foveal development are also poorly understood, but may be regulated by ATF6 and other yet to be determined genetic cues and molecules. Clinically, however, we do know, that many types of inherited retinal degenerations (IRD) ranging in severity from Leber congenital amaurosis (LCA) to retinitis pigmentosa (RP) have or develop macular and foveal atrophy, also misnamed as macular coloboma.

Currently, 92 *CRX* mutations have been reported (HGMD, professional 2017)^4^, consisting mostly of heterozygous missense/nonsense mutations, small deletions, duplications and insertions giving rise to a complicated range and severity spectrum of retinal phenotypes, extending from the mild Benign Concentric Annular Dystrophy and autosomal dominant macular dystrophy, to the much more severe LCA, RP and CRD. The overwhelming number of reported cases is dominant. Rarely, homozygous and compound heterozygous (autosomal recessive inheritance) mutations have been reported in *CRX*^4^.

Four exons make up *CRX* (3 coding), producing a 299 amino acid protein, with strong homology to *OTX1* and *OTX2*, two other retinal development genes/transcription factors. CRX is made up of three important protein domains; a homeodomain, which binds to DNA of retinal genes, a WSP motif with unknown function, and an OTX tail at the carboxyl terminus also of unknown function. In all cases, it is predicted that an abnormal CRX protein is produced; because nonsense mediated decay is avoided in the terminal exon of *CRX*, where the frameshift and nonsense mutations are located^4,5^. Several mouse models (CRX^rip/+^ and others)^6,7^, plus the location and heterozygosity of the human *CRX* mutations suggests that haploinsufficiency of *CRX* is not, in itself, disease-causing, although haplo-insuffiency in other transcription factors has been documented^4,5^. Therefore, current thinking is that a mutant *CRX* allele is both nonfunctional and expressed, such that the abnormal CRX protein partly interferes with the normal one expressed from the other allele^5^. The mechanism of disease would therefore be dominant negative or gain of function.

In this study we have the unique opportunity to test the haplo-insuffiency versus the gain of function hypotheses for the *CRX* associated disease mechanism. Here we describe a complete, homozygous deletion of *CRX* in three affected LCA patients with significant macular colobomas. The LCA patients thus have nullizygosity and unexpectedly, we found mild phenotypes in the carrier parents, including mild foveal and photoreceptor abnormalities. The carrier parents thus have haplo-insufficiency but do not develop LCA. Our findings suggest a novel mechanism of disease due to *CRX*, with complete loss of function.

## Methods

**Figure 1:**
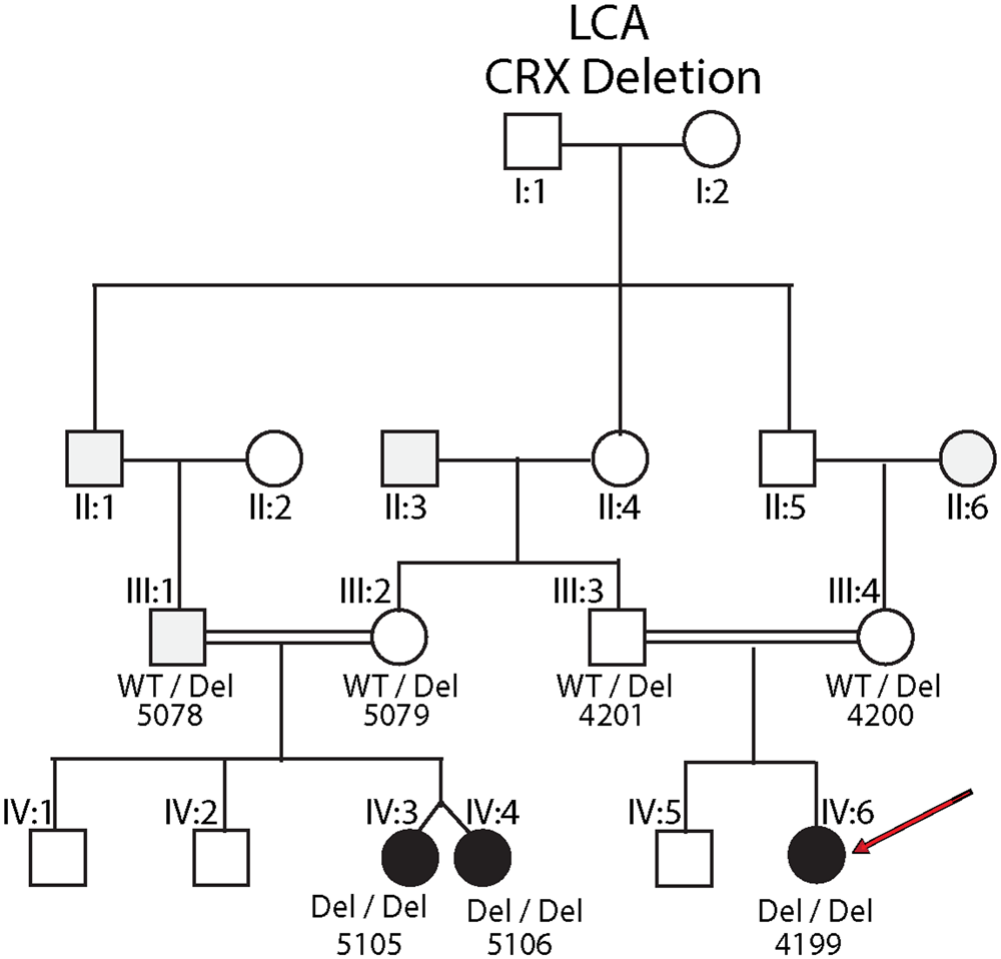
Pedigree of the Lebanese LCA family with consanguinity.

Our study consists of three children with LCA and their four parents (Fig. 1). Informed consents were obtained. The protocol and consent forms were approved by the MUHC RI REB and adhered to the tenets of Helsinki. The three children underwent standard paediatric ophthalmic evaluations, including visual acuity (VA) measures (by Snellen and Teller), fixation behaviour, slitlamp evaluations, dilated indirect ophthalmoscopy, and when possible *in vivo* retinal microscopy by ocular coherence tomography (OCT)(Heidelberg Inc), ERG and fundus photography. We then performed VA, OCT and fundus photography of the parents. Venous blood was taken of the 7 members (EDTA tubes) and genomic DNA was extracted from blood lymphocytes according to the kit protocol. The proband was originally sequenced by our inherited retinal dystrophy panel (www.molecularvisionlab.com). All genes on the panel were negative but an uncommon gap was identified in the *CRX* gene. We hypothesized a large CRX deletion. A homozygous deletion of *CRX* was confirmed by Quantitative PCR (qPCR). qPCR analysis was performed using the ABI TaqMan Copy Number Assay and CopyCaller Software. qPCR primers for the *CRX* gene, namely Hs03032227_cn (exon1) and Hs02743809_cn (exon4) were used for amplification and detection. RNAseP was used as a reference. The relative copy number was calculated. To define breakpoint of the *CRX* deletion, array CGH analysis was performed. DNA extracted from blood was analyzed using a comparative genomic hybridization (CGH) array from OGT (Eye gene array v2). Array data was analyzed by using OGT software CytoSure. The deletion was identified as arr[hg19] 19q13.33(48,313,541–48,348,194)x0. Unfortunately, the density of probes in the array was low outside the *CRX* coding regions. The left intact probe was 48,292,340–48,292,399 and the right intact probe was 48355017–48355076. Long range PCR was performed using forward primer from the 5′ region to the right intact probe and reverse primer from the 3′ region to the left intact probe. Next generation sequencing library was prepared from the long-range PCR product using One-tube NGS library preparation kit (Centrillion Technologies) and MiSeq (Illumina). We tested the deletion for co-segregation through the pedigree (Fig. 1).

## Results

**Figure 2:**
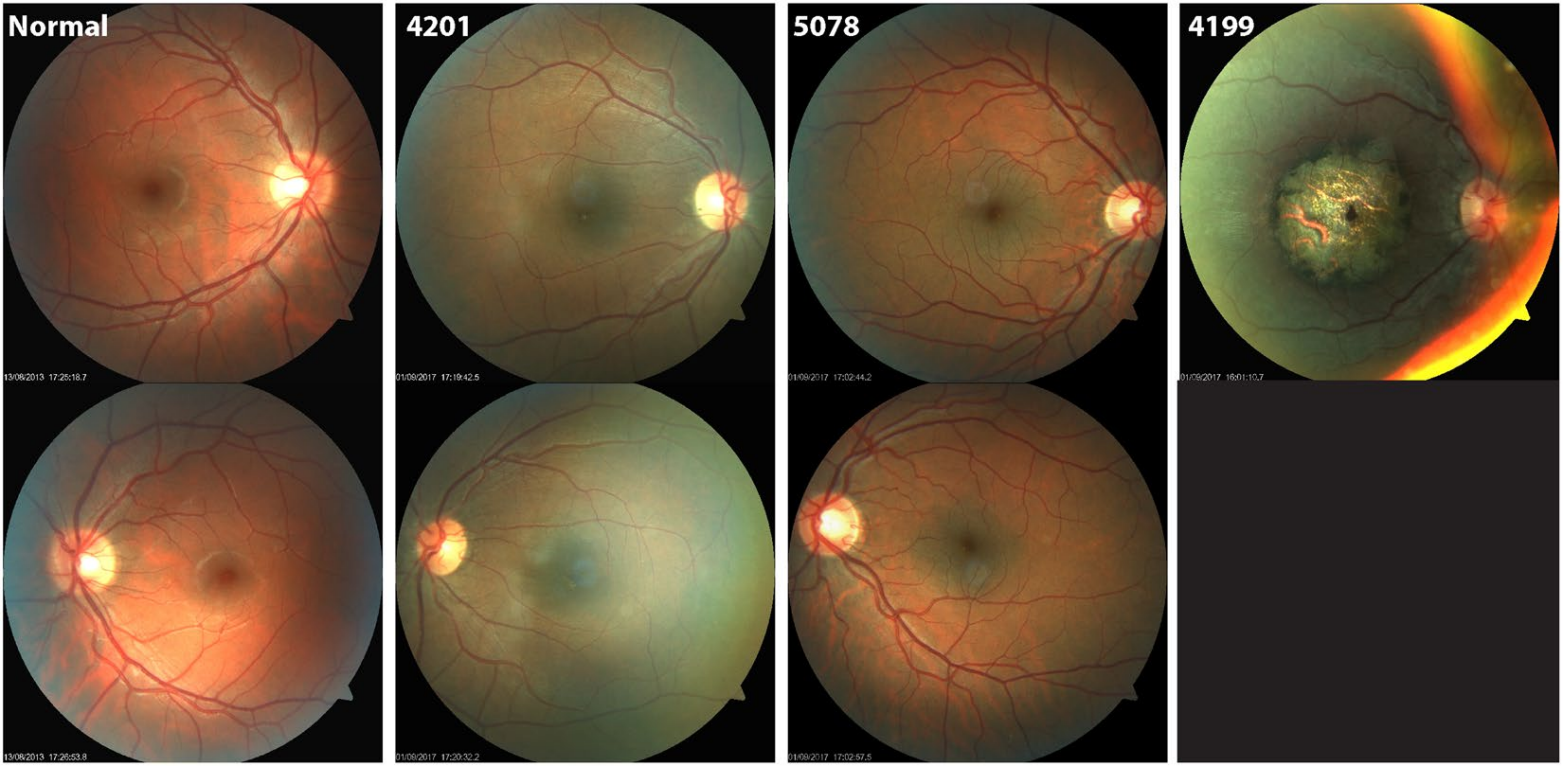
Color retinal photos of the normal control, two carriers and one affected LCA patient. Clearly shown are the bowl like umbo and the circular regular FAZ in the normal. In the two carriers, the umbo is flat, and the FAZ is irregular. In the LCA patient, a large macular coloboma is evident.

The proband (MOGL 4199) (Fig. 1) comes from a consanguineous Lebanese family that settled in Montreal, Quebec and was diagnosed with LCA at 1 month of age. She had no fixation at birth. At age 2, she was found to have some vision on exam in the light (but no vision in the dark), nystagmus, myopia and a severe macular coloboma (Fig. 2). OCT was not possible. The two identical cousins also had LCA with similar vision but with +5.00 hyperopia OU, manifest latent nystagmus and severe macular colobomas. One cousin developed the Coat’s reaction (exudative retinal detachment).

**Figure 3:**
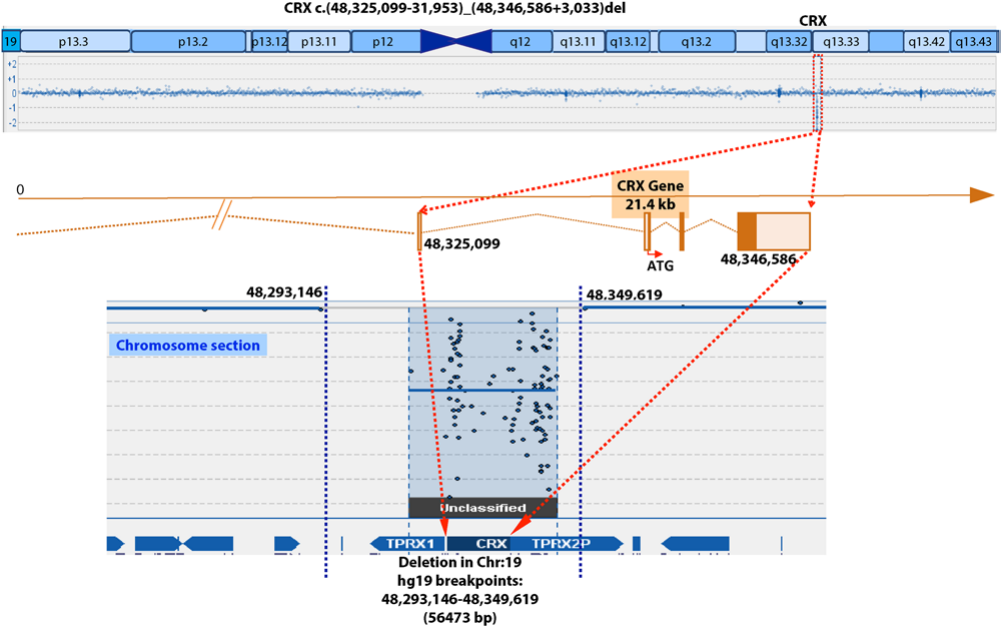
The chromosomal location of CRX, the CRX deletion, the CRX gene and exon structure, and the deletion breakpoints are shown.

The parents reported normal vision and had never visited Ophthalmology for themselves. Genetic analysis of 221 retinal genes in our array eye gene list (molecularvisionlab.com) on MOGL 4199 identified a break in *CRX* (Fig. 3). Long range PCR was performed using a left intact probe 48,292,340–48,292,399 and a right intact probe 48,355,017–48,355,076. We identified a 56,473 base pair (bp) deletion (Fig. 3) involving the entire *CRX* gene plus both flanking genes, with breakpoints hg version-19 chr19: 48,293,146–48,349,619. The homozygous deletion cosegregated with the LCA phenotype, as all three affected children harbored it. Also the four parents were heterozygous for the CRX deletion (Fig. 1). The LCA phenotypes of the three children are not different from the “typical” LCA phenotype or the specific CRX-associated phenotypes reported.

We then hypothesized that the carrier parents exhibit mild subclinical foveal or retinal abnormalities due to haplo-insufficiency of the *CRX* gene product. We thus brought the four parents back for evaluation by VA, OCT and fundus photography. All parents had 20/20 VA OU and no complaints or visual symptoms. Surprisingly, two of the parents were clearly not normal. Figure 2 shows the normal retinal appearance (OD and OS) of a normal control, contrasting with the retinas of the two fathers (MOGL 4201 and 5078). The bowl like (“pit”) appearance of the foveal umbo is not present in the two carriers. In the normal control, the foveal avascular zone (FAZ) is surrounding the foveal center in a circular fashion. However, in the carriers the FAZ appears abrogated. In MOGL 4201 the FAZ appears incomplete and non-circular, while in MOGL 5078, the FAZ is much smaller than normal and there is increased vascular branching. Also, MOGL 4201 has subtle lesions OU in the foveal center. The two carrier mothers (MOGL 4200 and 5079 in Fig. 1) appear to have normal umbos and FAZ, (data not shown).

**Figure 4:**
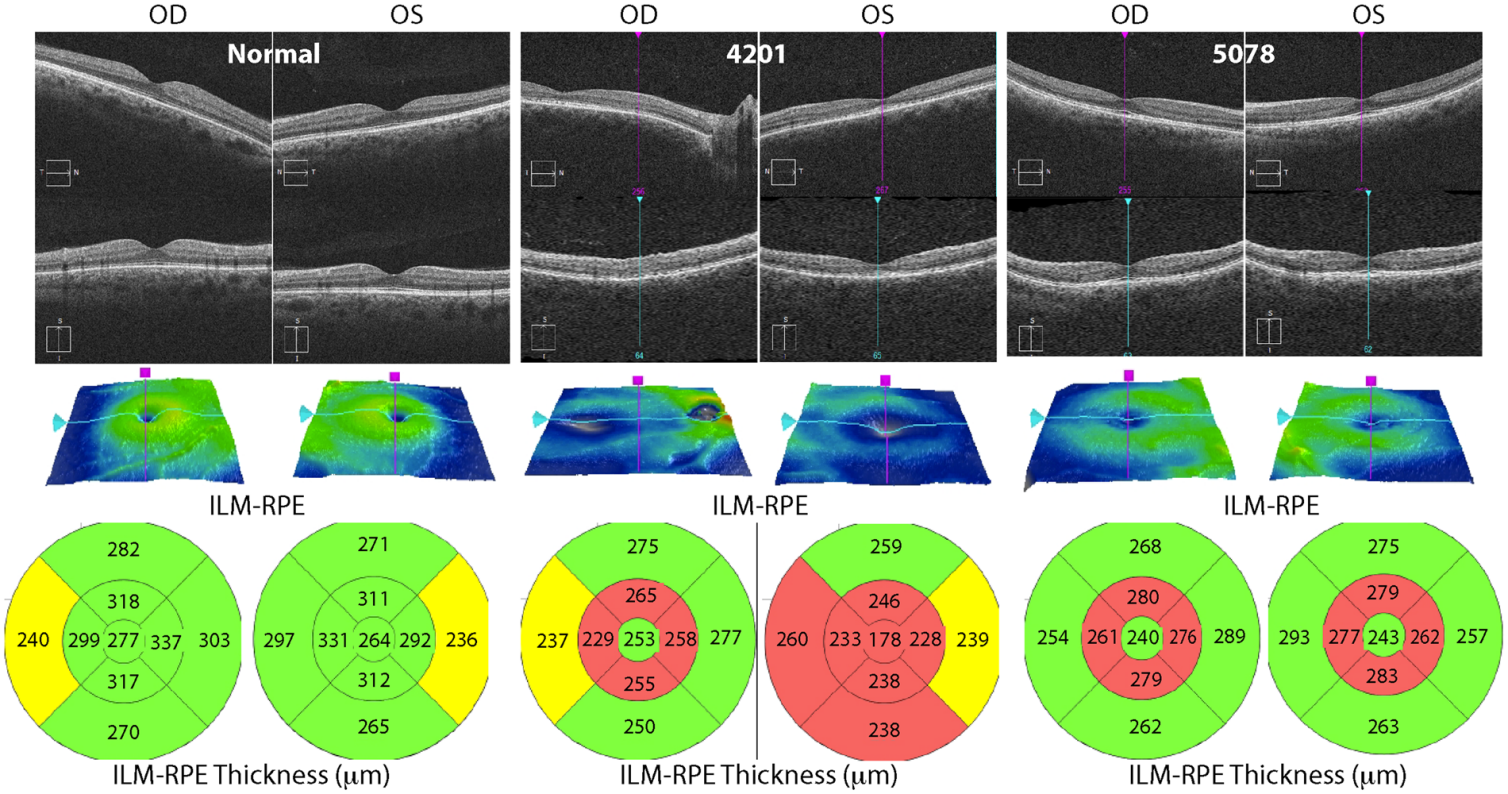
En face, EZ on OCT and thickness measurements (Heidelberg) are shown for a normal control and two carriers. Clearly the carriers have flattened umbos (en face), interrupted EZs and thinned foveal centers and parafoveal regions.

The *in vivo* retinal and foveal architecture were then studied by OCT (Heidelberg Inc) in a normal control and both MOGL 4201 and 5078, which had the abnormal foveal appearance on fundus photography. For all three subjects we performed horizontal and vertical scans through the foveas for OD and OS, and measures of the internal limiting membrane (ILM) to retinal pigment epithelium (RPE) distance (comprising the entire retinal thickness) in the foveal center and the parafoveal area and reported in μm (Fig. 4). We also performed *en-face* imaging (in color).

The normal control on the left (Fig. 4) shows the normal umbo (pit) formation (in the *en-face* color figures), the intact inner segment/outer segment junctions of the photoreceptor cells (IS/OS junction line) (aka the Ellipsoid Zone, EZ) (in the black and white OCT) and thickness in the fovea and parafovea, ranging from 264–270 μm in the center to 292–331 μm in the parafoveal ring surrounding the fovea.

In the carrier MOGL 4201 however, the *en-face* images are highly abnormal, and show that there is a marked flattening of the umbo (Fig. 4). The foveal pit is not normally developed. The OCT then shows that the EZ is not intact; it is interrupted in the center and especially in the parafoveal area (Fig. 4), indicating the photoreceptor IS and OS are interrupted. Also, there is marked foveal thinning as the foveal center was measured to be 178 μm (OS), an almost 100 μm difference with the normal control, (OD was not measured in the foveal center). Finally, the parafoveal thinning was documented and measured at 228–246 μm, a difference with the normal control of almost 100 μm as well.

**Figure 5:**
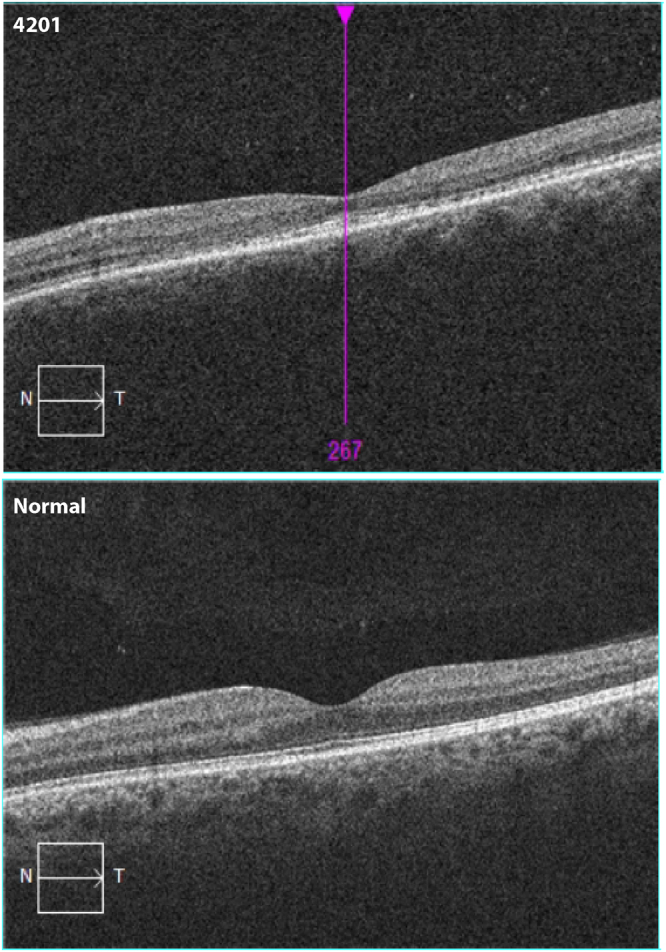
Detailed EZ images of a control and a carrier showing the EZ interruptions.

Carrier patient MOGL 5078 has similar abnormalities but milder (Fig. 4). The OCT and measurements of the fovea are within normal limits for the carrier female mothers (data not shown). Figure 5 shows the detailed foveal and parafoveal retinal layering and architecture in MOGL 4201 compared to normal in the horizontal plane, illustrating the marked EZ abnormalities.

## Discussion

We show in this study that a complete homozygous deletion of *CRX* causes LCA and a heterozygous *CRX* deletion can cause significant but subclinical inner and outer foveal abnormalities, but not LCA. Thus nullizygosity of *CRX* causes LCA, but haploinsufficiency of *CRX* does not cause LCA. However, haploinsufficiency can cause unexpected foveal abnormalities without disturbing visual acuity. Our homozygous complete *CRX* deletion causes a nullizygous state, the first described in human and is recapitulated in the *CRX*^*−/−*^ mice^8^. The *CRX*^*−/−*^ nullizygous mice do not elaborate photoreceptor outer segments and then degenerate and die rapidly. This strongly suggests that the nullizygous mice have a developmental retinal abnormality that leads to degeneration. The heterozygous *CRX*^−/+^ mice photoreceptors elaborate shorter than normal outer segments. Our parent carriers (2 out of 4) likely have shorter outer segments than normal, like the mice, as we found IS/OS junction (EZ) abnormalities on OCT, showing outer retinal changes. Our human subjects also have other foveal inner segment, structural abnormalities, including a very shallow umbo (pit), parafoveal photoreceptor loss and a poorly developed FAZ. We therefore show that *CRX* plays a role in foveal development. Previously, the molecular mechanisms of foveal development have been elusive. The triggers for the pit formation, centrifugal migration of three inner retinal layers, the FAZ and rod free zone formation, plus the packing of cones from 25,000/mm^2^ at birth to 200,000/mm^2^ at age 4–5 years old are poorly understood. Very recently, Da Silva and Cepko revealed that localized suppression of retinoic acid signaling regulates high-acuity area retina formation through a growth factor, Fgf8. Retinoic acid therefore likely controls human foveal development^9^.

The foveal abnormalities we found in humans (carriers) can be developmental or acquired and progressive. It is more likely that they are developmental and that the haploinsufficiency of *CRX* causes the development of the fovea to be arrested. The recapitulation in the *CRX*^−/+^ mice, the lack of vision loss, the lack of progression (despite their ages in the 40 s and 50 s), and the lack of atrophy, degeneration and pigmentation, in the human subjects here studied, make the acquired/progressive hypothesis less likely.

*CRX* is expressed in the nuclei of photoreceptors and the CRX protein recognizes the promotor regions of many retinal photoreceptor genes as they require *CRX* for normal expression, in conjunction with *NRL*, another transcription factor. The *CRX*^−/−^ mouse has greatly reduced levels of expression of many retinal genes^10^.

The CRX protein comprises three highly conserved domains, the homeobox, the WSP and the OTX domains. The homeobox has DNA binding activity, while the function of the WSP and OTX domain remain unknown.

Rivolta *et al*.^4^, first noted and reported a peculiar distribution of *CRX* mutations across the gene/protein. Missense mutations preferentially affect homeobox residues, while frameshift mutations leading to premature stops are found in the last exon, which encodes OTX domain^4^. Last exon truncating mutations avoid RNA mediated decay and lead to stable, shortened and (dys) functional CRX protein. In this way both missense and frameshift mutations cause CRX mutants that retain its ability to interact (interfere) with wildtype CRX in heterozygotes. Thus both categories of mutations would act in a dominant negative fashion^4^.

Haplo-insufficiency would thus not play a role in CRX-induced retinal degeneration under this model of action. *CRX*^−/+^ mice corroborate this point of view, as they do not develop LCA^10^. Our study results also agree with these points of view, as our heterozygous single *CRX* deletion does not lead to LCA, while our double *CRX* deletion does. Haploinsufficiency therefore does not lead to LCA, but nullizygosity of *CRX* does. *CRX* haploinsufficiency leads to a previously unreported mild but significant foveal development abnormality phenotype. Swaroop *et al*. were the first to report a homozygous missense mutation in autosomal recessive LCA, with mild carrier findings in the central retina, but *in vivo* retinal architectural studies of the carriers by OCT was not yet available in 1999. Their mutation is likely an example of gain of function, while our proposed mechanism is loss of function in both affected and carriers^11^. We found the foveal abnormalities in two males but not in two female carriers. This gender difference may be due to chance, or gender and gene specific differences beyond the scope of this study. By reporting on the first complete homozygous *CRX* deletion and the resulting nullizygous state, we have thus identified a novel *CRX* mediated disease mechanism. Nichols *et al*., in *in vitro* CRX and NRL binding and localization studies of two CRX mutations, confirm that haplo-insufficiency of *CRX* may not be pathogenic, and that various dominant negative or “dominant negative like” effects may play a role^12^.

We thereby confirm that haplo-insufficiency of *CRX* does not cause LCA, and that the previously reported *CRX* mutations likely act mainly through a dominant negative or a “dominant negative like” mechanism. *CRX* haplo-insufficiency does however affect foveal development.

## Electronic supplementary material

Supplementary Information
