# Supplementary material for: A complete, homozygous CRX deletion causing nullizygosity is a new genetic mechanism for Leber congenital amaurosis
Source: Sci Rep. 2018 Mar 22;8:5034. doi: 10.1038/s41598-018-22704-z (PMC5864841; doi:10.1038/s41598-018-22704-z)
Supplement: Supplementary file 1 — Supplementary Information [file 41598_2018_22704_MOESM1_ESM.docx]

A complete, homozygous *CRX* deletion causing nullizygosity is a new genetic mechanism for Leber congenital amaurosis.

Ibrahim MT; Alarcon-Martinez T, Lopez I, Fajardo N,

Chiang J*, and Koenekoop RK*.

Appendix

Array eye gene list:
ABCA4, ABHD12, ADAM9, AHI1, AIPL1, ALMS1, ARL13B, ARL2BP, ARL6, ATP13A2, B3GALTL, BBIP1, BBS1, BBS10, BBS12, BBS2, BBS4, BBS5, BBS7, BBS9, BCOR, BEST1, BMP4, C1QTNF5, C2orf71, C5orf42, C8orf37, CA4, CABP4, CACNA1F, CACNA2D4, CAPN5, CC2D2A, CDH23, CDH3, CDHR1, CEP290, CEP41, CERKL, CHM, CIB2, CLN3, CLN5, CLN6, CLN8, CLRN1, CNGA1, CNGA3, CNGB1, CNGB3, CNNM4, COL11A1, COL11A2, COL2A1, COL4A1, COL9A1, COL9A2, CRB1, CRX, CRYBA4, CTSD, CYP1B1, DFNB31, DHDDS, DTHD1, EFEMP1, ELOVL4, EMC1, EYS, FAM161A, FLVCR1, FOXC1, FOXE3, FRAS1, FREM1, FREM2, FSCN2, FZD4, GNAT1, GNAT2, GPR125, GPR143, GPR179, GPR98, GRIP1, GRK1, GRM6, GRN, GUCA1A, GUCA1B, GUCY2D, HARS, HCCS, IDH3B, IMPDH1, IMPG2, INVS, IQCB1, KCNJ13, KCNV2, KCTD7, KIAA1549, KIF7, KLHL7, LCA5, LRAT, LRIT3, LRP5, LZTFL1, MAK, MERTK, MFN2, MFRP, MFSD8, MKKS, MKS1, MYO7A, MYOC, NDP, NMNAT1, NPHP1, NPHP3, NPHP4, NR2E3, NRL, NYX, OCA2, OFD1, OPA1, OPA3, OPTN, OTX2, PAX6, PCDH15, PDE6A, PDE6B, PDE6C, PDE6G, PDE6H, PDZD7, PEX7, PHYH, PITPNM3, PITX2, PITX3, PLA2G5, PPT1, PRCD, PROM1, PRPF3, PRPF31, PRPF6, PRPF8, PRPH2, RAB28, RAX2, RBP3, RBP4, RD3, RDH12, RDH5, RGR, RGS9, RGS9BP, RHO, RIMS1, RLBP1, ROM1, RP1, RP2, RP9, RPE65, RPGR, RPGRIP1, RPGRIP1L, RS1, SAG, SDCCAG8, SEMA4A, SIX6, SLC24A1, SLC45A2, SMOC1, SNRNP200, SOX2, SPATA7, STRA6, TCTN1, TCTN2, TCTN3, TIMM8A, TIMP3, TMEM126A, TMEM216, TMEM237, TMEM67, TOPORS, TPP1, TRIM32, TRPM1, TSPAN12, TTC21B, TTC8, TULP1, TYR, TYRP1, UNC119, USH1C, USH1G, USH2A, VAX1, VCAN, VSX2, WDPCP, WT1, ZNF423, ZNF513, WFS1, CYP4V2, MTTP, OAT

.
